# Burnout syndrome et ses facteurs chez les médecins de deux centres Hospitalo-Universitaires d’Antananarivo

**DOI:** 10.11604/pamj.2018.31.63.11123

**Published:** 2018-09-27

**Authors:** Aurélia Rakotondrainibe, Harifetra Mamy Richard Randriamizao, Noro Seheno Ratsimbazafy, Yvon Mong-Gine, Catherine Nicole Rakotoarison, Felantsoa Auberlin Rakototiana, Marie Lydia Agnès Ravalisoa

**Affiliations:** 1 Faculté de Médecine d’Antananarivo, Université d’Antananarivo, Madagascar; 2 Direction de la Cellule d’Appui à la Mise en Œuvre de la Couverture Santé Universelle, Madagascar

**Keywords:** Epuisement professionnel, Madagascar, MÃ©decins, MÃ©decine du travail, Professional exhaustion, Madagascar, physicians, occupational medicine

## Abstract

La profession médicale est un métier stressant, pouvant engendrer un syndrome d'épuisement professionnel ou *burnout syndrom* (BOS). Le but de cette étude était de déterminer les degrés du BOS (faible, moyen, élevé) de par ses dimensions et les facteurs liés à l'activité professionnelle du médecin qui lui étaient corrélés. Il s'agit d'une étude transversale, en 2012, par auto-questionnaire, auprès des médecins du Centre Hospitalier de Soavinandriana et du Centre Hospitalier Universitaire, Joseph Ravoahangy Andrianavalona. Des tests de corrélation et de régression linéaire ont été effectués (SigmaStat^®^ 3.5). Le taux de réponse à l'enquête a été de 47,1% sur 138 médecins hospitaliers. Le nombre de dossiers retenus était de 48. La population de l'étude était à prédominance masculine (sex ratio: 1,8) avec un âge médian de 37 [25-59] ans. Les internes de spécialité et les médecins assistants représentaient 56,3% de la population. Selon l'ancienneté 16,7% étaient dans le métier depuis moins d'un an. Le *burnout syndrom* a été observé dans 51,2 % des cas avec un degré élevé pour 4,2% des médecins. Le titre avait une corrélation significative avec le syndrome d'épuisement professionnel et son degré (p=0,0142 et p=0,0362), notamment l'épuisement émotionnel (p=0,0414). L'apparition du BOS n'était ni corrélé avec l'ancienneté du médecin ni avec le secteur d'activité. Le BOS existe en milieu hospitalier, surtout lié au titre du médecin. Il est essentiel de le diagnostiquer au plus tôt pour en éviter ses conséquences délétères.

## Abstract (en)

Medical profession is stressful and can cause professional exhaustion syndrome or Burnout syndrome (BOS). This study aims to determine Burnout syndrome levels (low, medium, high) by its dimensions and by causing factors related to physicians’ professional activity. We conducted a cross-sectional study based on self-administered questionnaire to practicing physicians in Soavinandriana Hospital Center and Joseph Ravoahangy Andrianavalona University Hospital in 2012. Correlation and linear regression analyses were made (SigmaStat^®^ 3.5). Out of 138 hospital physicians, questionnaire response rate was 47.1%. Forty eight medical records were selected. The study population was male-dominated (sex ratio: 1.8) with a median age of 37 [25-59] years. Physicians-in-training and assistant physicians accounted for 56.3% of the study population. According to the seniority of the physicians, 16.7% had worked for less than 1 year. Burnout syndrome was found in 51.2% of physicians, of whom 4.2% had high syndrome levels. Occupational status was significantly correlated with Burnout syndrome and with its level (p=0.0142 and p=0.0362), including emotional exhaustion (p=0.0414). BOS wasn’t related with physician seniority and sector of activity. BOS in hospital environment is mainly related to occupational status. Early diagnosis is essential to prevent its deleterious effects.

## Introduction

Le syndrome d'épuisement professionnel ou *burnout syndrom* (BOS) est une pathologie qui peut survenir dans les professions à visée sociale et communautaire [1]. La profession médicale est très stressante ; le stress chronique qui lui est lié peut engendrer le BOS, et avoir un effet délétère sur la qualité des soins [2, 3]. Ce syndrome peut avoir un impact négatif sur la qualité de vie des professionnels, tant au niveau psychologique que physique [4]. Le *Maslach Burnout Inventory* (MBI) en 22 items, est la perspective dominante et l'instrument de référence pour mesurer le BOS [4-6]. Les spécialités affectées par cette pathologie et les proportions du BOS chez les médecins sont différentes suivant les études, allant de 33,0% à 78,1% [2, 6-8]. Les généralistes tout autant que les spécialistes sont enclin à avoir un syndrome d'épuisement professionnel [9-11]. Nous avons réalisé une étude, auprès des médecins de deux centres hospitaliers, à activité chirurgicale, d'Antananarivo - Madagascar, dans le but de déterminer les degrés du BOS (faible, moyen, élevé) de par ses dimensions: l'épuisement émotionnel (EE), la dépersonnalisation (D), l'accomplissement professionnel (AP) et les facteurs liés à l'activité professionnelle du médecin qui étaient corrélés à ce BOS.

## Méthodes

Il s'agit d'une étude transversale, par enquête sur auto-questionnaire, auprès des médecins de deux centres hospitaliers d'Antananarivo - Madagascar: le Centre Hospitalier de Soavinandriana (CENHOSOA) et le Centre Hospitalier Universitaire, Joseph Ravoahangy Andrianavalona (CHU JRA). L'enquête été réalisée en Juin 2012. Les médecins concernés par l'étude travaillaient dans le milieu chirurgical, notamment en service de chirurgie, des urgences chirurgicales et en anesthésie réanimation A titre d'état des lieux, la charge de travail en 2012 était représentée par une médiane de patients opérés par mois, au CENHOSOA de 34 pour les interventions en urgence et 62 pour les interventions programmées, pour le CHU JRA de 48 interventions urgentes et 165 blocs programmés. Le nombre de questionnaires distribués en mains propres était en fonction du nombre de médecins des différents services, ayant accepté de participer à l´enquête. S´agissant d´auto-questionnaire, le délai prédit de remplissage du questionnaire était de 15 jours et les fiches remplies étaient reprises au sein même du service par l´enquêteur. L'auto-questionnaire était anonyme, sans mention de l'identité de l'enquêté. Le questionnaire comprenait deux pages : la première comportait, outre la demande de participation, les informations personnelles du médecin enquêté ; parmi lesquelles, son genre, son année de naissance, son titre, le centre hospitalier d´exercice, le secteur d´activité et l´ancienneté dans le travail. La deuxième page était réservée à l'évaluation du BOS selon le MBI, de 22 items comme détaillée par Maslach [5], dont les réponses sur la fréquence étaient à cocher. Les questionnaires remplis étaient parvenus et analysés dans les quinze jours à deux mois suivant leur distribution. L'échantillon d´étude concernait les médecins qui ont accepté de participer à l´étude. Les réponses au questionnaire ont été enregistrées et analysées par SigmaStat^®^ 3.5. Toutes les fiches recueillies, auprès des médecins, de la population cible, ayant accepté de participer à l'enquête ont été analysées. Le principal critère d'exclusion était le manque de réponse(s) dans le MBI ; le *burnout syndrom* n'a pas été analysé lorsqu'une réponse ou plus étaient manquantes. Les paramètres analysés étaient : les critères démographiques (âge, genre, titre hospitalo-universitaire), les critères liés à l'activité professionnelle (ancienneté, centre hospitalier d´exercice, secteur d´activité (chirurgie, anesthésie-réanimation, urgences chirurgicales)) et ceux liés au BOS (sa présence ou non, son degré ainsi que les caractéristiques de ses dimensions). Les variables qualitatives ont été exprimées par leurs fréquences ; les variables quantitatives ont été exprimées à l´aide de leurs médianes avec extrêmes (minimum et maximum). Le critère de jugement primaire a été le syndrome d'épuisement professionnel (de par ses degrés et ses dimensions) chez les médecins et le critère de jugement secondaire a été la relation entre le BOS et les facteurs liés à l'activité professionnelle des médecins. La recherche des corrélations a été effectuée par l´intermédiaire du test de corrélation de Spearman et des tests de régression linéaire ont été effectués. Une valeur de p inférieure à 0,05 a été considérée comme significative.

## Résultats

**Figure 1:**
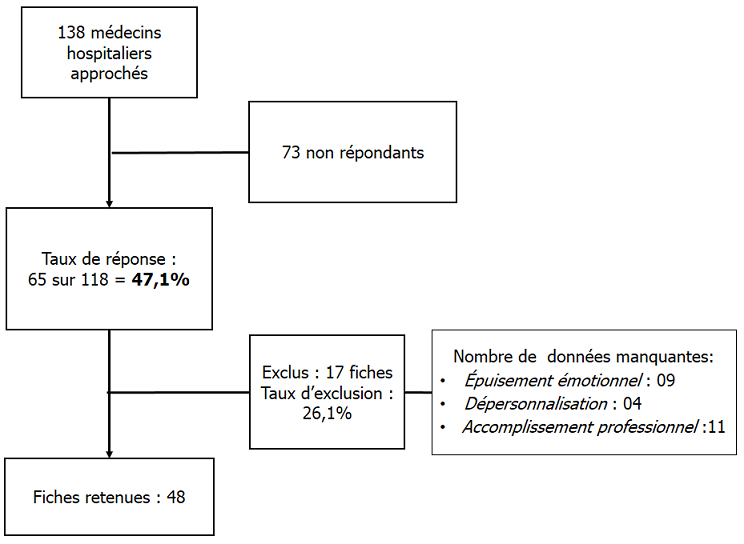
Analyse de l’enquête

**Figure 2:**
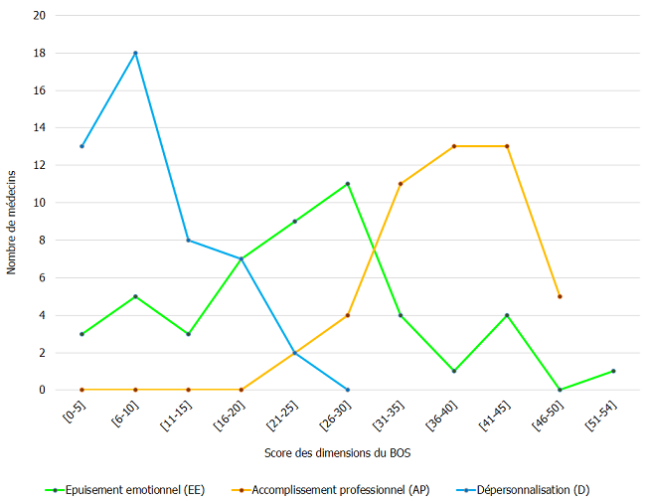
Scores des dimensions du BOS

**Figure 3:**
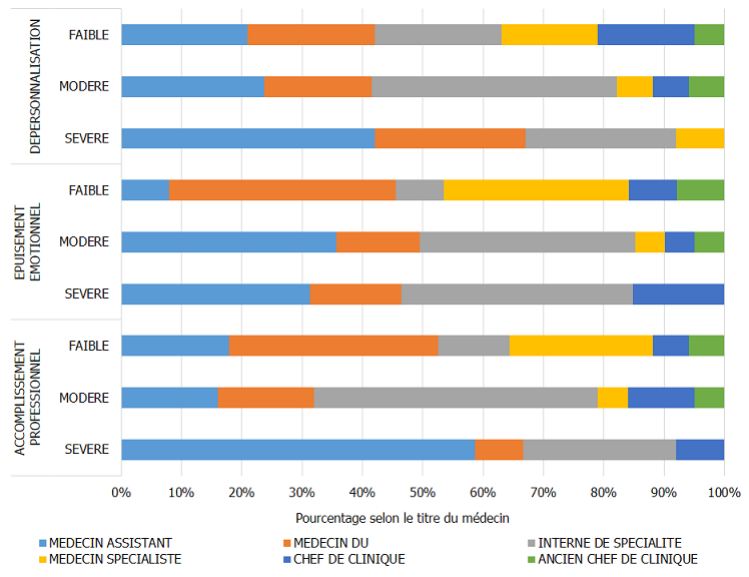
Degré de sévérité des dimensions du BOS et titre

**Figure 4:**
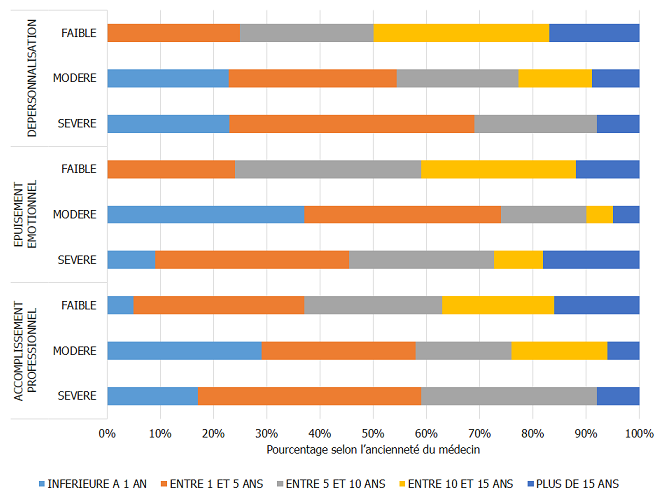
Degré de sévérité des dimensions du BOS et ancienneté

**Figure 5:**
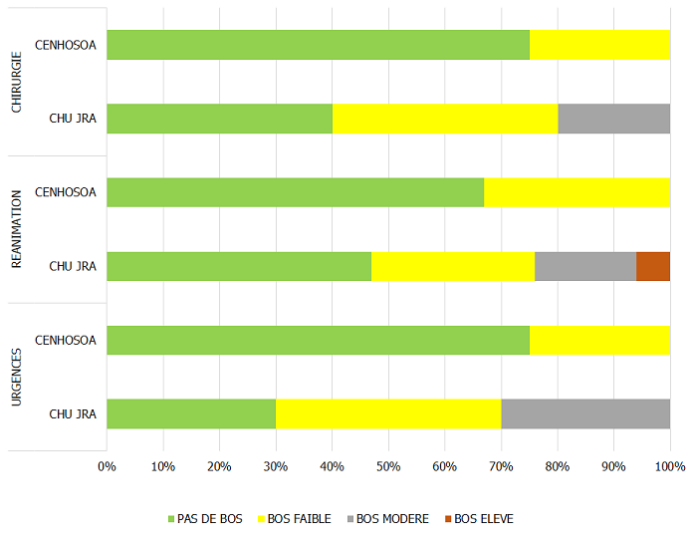
Présence et sévérité du BOS selon le secteur d’activité

Le taux de réponse à l'enquête a été de 4^7^,1% sur 13^8^ médecins hospitaliers (Tableau 1), le taux d'exclusion était de 21,6%. Le nombre de dossiers retenus était de 48 (Figure 1). La population de l´étude était à prédominance masculine avec un sex ratio de 1,8 (64,6% de médecins du genre masculin et 35,4 % de médecins du genre féminin). L´âge médian des médecins était de 37 [25-59] ans. Les internes de spécialité (14) et les médecins assistants (13) étaient les plus prépondérants dans la population, représentant 56,3%. Selon l'ancienneté, 16,7% étaient dans le métier depuis moins d'un an, 33,3% avaient entre un et cinq ans d'ancienneté, 25,0% de cinq à 10 ans et 31,2% plus de 15 ans. Le burnout syndrom a été observé dans 51,2 % des cas. Dans le cas où le BOS était présent, il était de degré faible (31,3 %), moyen (16,7%) et élevé (4,2%). Le degré du BOS présents chez les médecins hospitaliers en milieu chirurgical était fonction du degré d´atteinte des dimensions de ce syndrome (Figure 2); l'accomplissement professionnel était faible (39,6%), modéré (35,4%), élevé (25,0%), de même, la dépersonnalisation et l'épuisement émotionnel étaient faibles (27,1% / 35,4%), modéré (45,8% / 39,6%) et élevés (27,1% / 25,0%). Nous avons recherché les corrélations du BOS par rapport aux caractéristiques professionnelles, notamment le titre du médecin, l'ancienneté dans le travail et le lieu d'exercice (secteur d'activité et centre d'exercice). Le titre avait une corrélation significative avec le syndrome d´épuisement professionnel (p=0,0142), de même avec le degré du BOS (p=0,0362). Portant sur les dimensions du BOS, l'épuisement émotionnel était significativement corrélé avec le titre des médecins (p=0,0414). L'accomplissement professionnel ainsi que la dépersonnalisation n'étaient pas significativement corrélés avec le titre du médecin enquêté, bien que cette dernière dimension ait été retrouvée dans des proportions assez importantes chez les médecins assistants et les internes de spécialité, allant de modérée à sévère (Figure 3). L´apparition du BOS ainsi que son degré de sévérité n´étaient pas corrélés avec l´ancienneté du médecin. Nous avons pu cependant constater que les médecins ayant une ancienneté entre un et cinq ans présentaient une forte proportion de dépersonnalisation, suivis des médecins exerçant depuis 5 à 10 ans (Figure 4). Plus le médecin gagnait en ancienneté, plus les valeurs de la dépersonnalisation allaient en diminuant de façon significative (R^2^=0,1 ; p=0,0261) ; alors que l´ancienneté avait tendance à faire diminuer l´EE et augmenter l'AP sans lien évident. L´apparition du BOS, son degré ainsi que le degré de chacune de ses dimensions n´avaient aucune corrélation avec le secteur d'activité: chirurgie, anesthésie-réanimation ou urgences (Figure 5). Il en a été de même avec le centre hospitalier où exerçait le médecin; outre le degré d´épuisement émotionnel était significativement corrélé avec le centre d´exercice (p=0,0266) et cet épuisement professionnel était plus prépondérant pour ceux qui travaillaient au CHU JRA (sur les 37 médecins répondants du CHU JRA, 45,9% présentaient un degré d'EE modéré et 27,0% un degré d'EE sévère).

**Tableau 1:** Participation selon le site

Centre hospitalier	Service	Nombre de médecins	Questionnaires remplis et remis	Exclus	Pas de participation
**CENHOSOA**	Réanimation	10	5	2	5
	Urgences chirurgicales	14	6	2	8
	Chirurgie générale et digestive	6	6	2	0
	Chirurgie générale et cardio-vasculaire	4	0	–	4
**Sous-total 1**	(n)	**34**	**17**	**6**	**17**
	**(%)**	**100,0**	**50,0**	**17,6**	**50,0**
**CHU JRA**	ATUR	26	13	3	13
	Réanimation chirurgicale	24	21	3	3
	Neuro-chirurgie	8	5	2	3
	Urologie	15	7	2	8
	Chirurgie viscérale	11	0	–	11
	Chirurgie thoracique	5	1	1	4
	Traumatologie adulte	5	0	–	5
	Chirurgie infantile	10	1	0	9
**Sous-total 2**	(n)	**104**	**48**	**11**	**67**
	(%)	**100,0**	**46,1**	**10,6**	**64,4**
**TOTAL**	(n)	**138**	**65**	**17**	**84**
	(%)	**100,0**	**47,1**	**12,4**	**60,8**

## Discussion

Dans notre étude, la fréquence du *burnout syndrom* était de 51,2 % des cas avec 4,2% des médecins qui présentaient un BOS sévère. Le titre du médecin évalué était corrélé avec ce syndrome d´épuisement professionnel. En termes d'ancienneté, cette dernière influençait sur la dépersonnalisation. Le secteur d'activité des médecins n'avait pas de corrélation avec le BOS et le centre d'exercice affectait l'épuisement émotionnel. La force de cette étude est d'avoir étudié un mal contemporain qui pourrait avoir un impact sur la qualité de travail du médecin. Outre le bien être du patient, il est essentiel de considérer celui du personnel soignant car de celui-ci dépend la qualité de son travail. Cependant, le taux de non-réponse dans cette étude était élevé, de l'ordre de 52,9%, pourtant les médecins que nous avons approchés n'étaient pas réticents à cette enquête. Néanmoins, la plupart d'entre eux n'étaient pas toujours disponibles, de par la charge de travail et l'urgence des soins dans la plupart des services concernés. Ce taux de réponse aurait pu être également être expliqué par le non vouloir d'un immiscion dans leur vie privée, malgré un questionnaire anonyme.

L´épuisement professionnel est défini comme un syndrome psychologique relatif à des facteurs de stress interpersonnels chroniques au travail [1]. La dimension de l´épuisement émotionnel représente une contrainte individuelle se référant à des sentiments d´être épuisé de ses ressources émotionnelles et physiques. La dépersonnalisation représente une réponse négative, un détachement à divers aspects du travail, la dimension du contexte interpersonnel de l´épuisement professionnel. L'accomplissement personnel diminué représente la dimension d´auto-évaluation de l´épuisement professionnel relative à des sentiments d´incompétence et un manque d´accomplissement et de productivité au travail [1]. Dans les deux centres à visée chirurgicale d'Antananarivo, Madagascar, nous avons pu constater qu'un peu plus de la moitié des médecins enquêtés (51,2%) présentaient un syndrome d'épuisement professionnel, parmi lesquels 4,2% d'entre eux avaient un degré élevé de BOS. Ce dernier était corrélé avec le titre hospitalo-universitaire du médecin, et cette relation était significative, notamment avec l'épuisement émotionnel. Comparés à la population générale, les médecins sont concernés à près de 37,9% par le BOS [12]. Le personnel médical est 1,72 fois plus exposé au BOS que le personnel non médical, avec un BOS sévère de l'ordre de 6,3% à 21,5% contre 12,5% [13, 14]. Le degré de sévérité des dimensions est variable : 29,5% à 37,9% d'épuisement émotionnel élevé, 15,7% à 29,4% avec niveau élevé de dépersonnalisation et 12,4% à 19,7% avaient un faible niveau d´accomplissement personnel [12, 14]. La sévérité du degré de ces dimensions est liée au stress entraînant une exposition 27 fois plus importante d'épuisement émotionnel, liée à la menace d'identité (3,2 fois plus d'exposition à une diminution de l'accomplissement professionnel) et liée aux deux entités exposant à la dépersonnalisation [15]. L'ancienneté dans le travail peut également influencer l'apparition du BOS [16]. Dans notre étude, l'ancienneté n'avait pas de lien significatif avec l'apparition du BOS; bien que cette composante en diminuait son intensité. Cette relation non significative a également été retrouvée dans une étude effectuée dans une université turque [9]. Par contre, certaines études ont retrouvé que le personnel plus âgé a un BOS plus faible par rapport aux jeunes, dans les trois dimensions [3, 14]. Les médecins expérimentés de 4 à 6 ans voire moins de 10 ans de pratique présentent un degré sévère plus important dans l'EE et la dépersonnalisation ainsi qu'un degré sévère de BOS [17, 18]. Une ancienneté dans le travail de l'ordre de 5 à 15 ans multiplierait par 5, le risque de BOS par rapport aux « jeunes recrues » de moins de cinq ans d'expérience, cependant ce syndrome diminue à plus de 10 à 15 ans d'ancienneté [14, 18].

Dans notre étude, l'apparition du BOS et sa sévérité étaient corrélées avec le titre du médecin enquêté, en particulier l'épuisement émotionnel. Les médecins assistants étaient plus concernaient par le BOS, dont les dimensions étaient les plus sévères. L'apparition du BOS varie selon le degré d'étude des travailleurs. Dans une étude au Yémen, aucune différence n'était constatée entre les spécialistes et les non-spécialistes dans l'apparition d'un BOS sévère [18]. Chou *et al*. [3] retrouve que ceux qui ont un diplôme plus élevé présentent un moindre degré de syndrome d'épuisement professionnel. Parmi les professions médicales, les infirmières et les médecins assistants en sont les plus concernés [3]. De plus, il est retrouvé que les médecins assistants et ceux qui ne concourent pas à poursuivre leur carrière hospitalo-universitaire avaient un plus fort taux de BOS élevé [19, 20]. L'apparition du BOS n'avait pas de corrélation significative avec le secteur d'activité des médecins dans notre étude. De même, Tomljenovic *et al*. [21] ne retrouvent pas de différence notable selon le secteur d'activité. Mais cela varie selon les études. Les médecins des hôpitaux étaient sujets au BOS dans 53,9% avec près de 10 à 11 fois plus de risque d'en être exposés par rapport aux médecins de famille [20]. Les médecins qui officient dans des services traitant des traumatisés, tels les urgentistes, les chirurgiens, risquent d'être exposé à une moindre compassion, une dépersonnalisation voire une diminution de l'accomplissement personnel [22, 23]. Selon les cas, les chirurgiens sont plus sujets au BOS par rapport aux praticiens de médecine interne et de sciences fondamentales bien que la chirurgie regroupe des spécialités variées et nombreuses [8, 9]. Ou inversement, les anesthésistes sont plus épuisés professionnellement que les chirurgiens, notamment dans les dimensions de l'épuisement émotionnel et de l'accomplissement personnel [10]. De même, les praticiens des services de soins intensifs, présentent un BOS sévère de l'ordre de 62% [24]. Dans une enquête française, les médecins urgentistes sont plus sujets au BOS, de l'ordre de 51,5% par rapport aux autres spécialités avec un degré plus élevé (33,0%). Toutes spécialités confondues, les urgentistes ont un risque 3,18 fois plus élevé de présenter un BOS [12]. Aussi, on pourrait dire que le BOS n'épargne aucune spécialité.

Le fait de travailler au sein d'un centre hospitalier universitaire n'influe pas sur le risque de survenue du syndrome de burnout dans notre étude. Dans la littérature, Doppia *et al*. [2] ainsi que Mion *et al*. [6] après avoir effectué des études sur une population d'urgentistes et d'anesthésistes-réanimateurs de centres hospitaliers universitaires français ont découvert respectivement un taux de prévalence du *burnout syndrom* de 42% et 62% mais n'ont trouvé aucun lien significatif entre le lieu de travail et le BOS. Une étude finlandaise retrouve que les médecins œuvrant en milieu hospitalier accusent d'un BOS modéré [11]. Bien que cette étude n'ait eu qu'un retour mitigé, détecter le BOS est nécessaire. En effet, le BOS dérive de l'accumulation de stress, d'un état dépressif ou mélancolique, ou encore d'un manque de sommeil, que peut engendrer la profession médicale [10, 22, 25]. De plus ce syndrome s'accompagne de nombreux effets autant psychiques que somatiques, voire des addictions; ce qui serait délétère dans leur prise en charge des patients [1, 22, 25]. Un des risques de survenue du BOS est l'absence de conditions optimales de travail et le risque d'erreurs médicales dans cette profession particulièrement stressante [17, 22]. Madagascar n'en est pas exempt. Un sondage plus conséquent serait plus intéressant, vu les limites de cette étude. Parmi celles-ci, le taux de retour faible et le type d'étude (transversale). Nous avions voulu voir la réceptivité des médecins à une enquête portant sur le BOS, mais, 47,1% des questionnaires ont été rendus. Cela aurait pu mésestimer les résultats obtenus et leur interprétation, que ce soit sur les paramètres démographiques, professionnelles voire le BOS et ses composantes. De plus, l'exclusion de dix-sept fiches dans laquelle au moins une question n'a pas eu de réponse aurait pu sur- ou sous-estimer ce syndrome d'épuisement professionnel. Par cette enquête transversale par laquelle les données étaient recueillies à un instant déterminé. Une dimension temporelle de l'étude, par une approche longitudinale, pourrait mieux cerner les facteurs temporels liés à ce syndrome (période de vacances, de fêtes, etc.).

## Conclusion

Déterminer la fréquence de ce mal contemporain dans les professions médicales est important. Malgré le faible taux de réponse, nous avons pu retrouver un taux non négligeable du syndrome d'épuisement professionnel. Ce dernier était particulièrement lié au titre du médecin, mais ne différait pas par rapport au service et au centre hospitalier où exerçait le praticien. Elargir l'enquête voire le diagnostic de cette pathologie en médecine du travail semble nécessaire, car sa survenue est délétère autant pour le médecin que les sujets qu'il prend en charge.

### Etat des connaissances actuelles sur le sujet

- Le *Burnout syndrom* est une réalité au sein des professions sociales telles que la médecine; sa fréquence est non négligeable.

### Contribution de notre étude à la connaissance

- Il s'agit des premières études qui étudient le BOS dans des centres hospitaliers malagasy, qui montre que ce syndrome existe bel et bien à Madagascar. La corrélation de ce syndrome surtout avec le titre du médecin a pu être déterminée.

## Conflits d’intérêts

Les auteurs ne déclarent aucun conflit d'intérêt.
